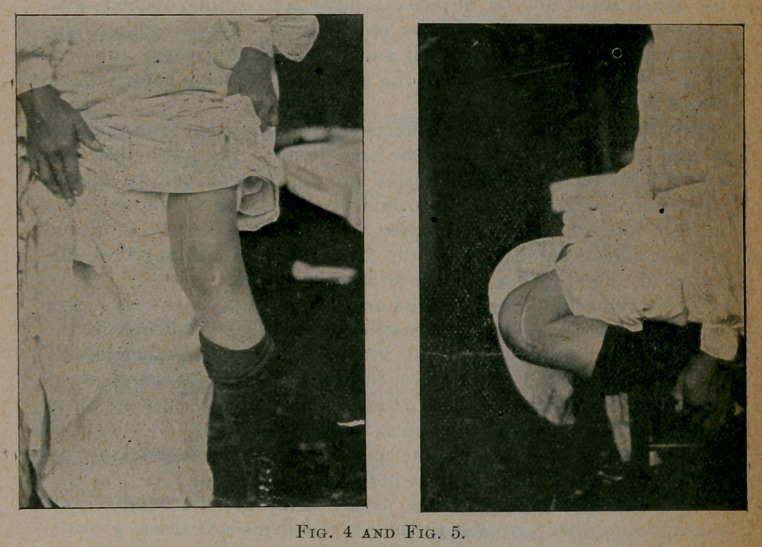# Conditions Found in Chronic Rheumatism of the Knees

**Published:** 1906-03

**Authors:** Michael Hoke

**Affiliations:** Atlanta, Ga.; Orthopedic Surgeon to the Presbyterian and Wesley Memorial Hospitals and the Tabernacle Infirmary, Clinical Professor of Orthopedic Surgery in the Atlanta College of Physicians and Surgeons


### CONDITIONS FOUND IN CHRONIC RHEUMATISM OF THE KNEES AND THE RELIEF AFFORDED BY OPERATIONS.*

BY MICHAEL HOKE M.D., ATLANTA, GA.

Orthopedic Surgeon to the Presbyterian and Wesley Memorial Hospitals and the Tabernacle Infirmary, Clinical Professor of Orthopedic Surgery in the Atlanta College of Physicians and Surgeons.

The cases reported in this paper are selected from seventy-nine cases of similar nature operated upon in the last five years. In each instance the patient complained of "chronic rheumatism,"

*Read before The Fulton County Medical Society.

and the condition in each instance had been called "chronic rheumatism" or "rheumatic gout" by the attending physician. For this reason and that I might point out the usefulness of operative work in these cases I have also used the term "chronic rheumatism."

The conditions found in the joints were variable; the degree or involvement of the osseous, synovial, fibrous and fatty tissues varied, but in all either polypi, fringes, spurs of bone, cartilaginous bodies, fibrous tissue thickening, fat tumors or some combination of these things were present; in each there was something which by its presence irritated and damaged the knee.

Many of the patients were suffering not so much from the continuance of the rheumatic inflammation as from the presence of these abnormal formations.

The treatment of many had been confined to administration of medicines. Those who were able made trips to Hot Springs and there took the baths. This method of treatment in some of the cases had been continued for years. Often such agencies are most potent; they are helpful always, but it is so manifestly wrong to try to relieve a joint in this way of things which can come out only through an incision and which must be removed in order to give relief, that I wish to urge you to examine these lame knees more carefully with the hands that you may find the newly formed pathological formations. Medicines, hot baths, tonics and a carefully regulated mode of living will cure many cases. Many cases will suffer almost as much and be almost as lame with such treatment as without it. These latter cases upon careful examination will be found to need surgical measures for additional relief. Surgery can not remove a diathesis, but most often if the joint be relieved of its pathological encumbrances, the diathesis can be controlled. I shall confine my remarks in this paper to the clinical and surgical aspects of the few cases selected from the larger number operated upon. These cases are selected to give a fair idea of the gross lesions found and a fair estimate of the relief possible, offered through operation.

CASE 1. Miss H. *Past History*—Dyspepsia, chronic constipation, often in bad health. Has had severe rheumatic pains in the past. Many joints were painful. One or two finger joints were swollen.

In October, 1903, she had an attack of what seemed to be acute rheumatism. Both knees were swollen. One or two finger joints were swollen and there were severe rheumatic pains over the body. Appropriate antirheumatic treatment was instituted by her family physician. She improved except in the right knee, which became flexed fifteen degrees. It could not be straightened. The joint was painful and swollen. The joint was very tender internal to the patella tendon and in the upper inner quadrant. She went to Hot Springs, Ark. She returned March first. Some of the swelling had subsided but the knee was still flexed and tender and she limped badly.

Operation March 29, 1904. The knee was opened on the inside. The capsule was thickened and the synovial surface injected. The glistening cartilege was altered in appearance by the presence of a threadlike network of blood-vessels. The ligamentum mucosum presented two enlarged, red, succulent tabs, which were removed. The fat in the pouch internal to the patella tendon was thickened and harder than normal. A part of this was removed.

The joint was thoroughly washed out and closed.

The site of the operation healed nicely. The patient's digestion suffered terribly from the anesthesia. There was a great deal of gas, nausea and more general pains. These pains continued for several months. Examination of the urine was negative.

Examination of the stomach and its contents showed some dilatation and a deficiency in hydrochloride acid. Electricity was given to the stomach wall for two months and a careful diet prescribed. The joint straightened perfectly with the postoperative use of caliper splints. There was no swelling in the knee after the operation. Gradually the digestion improved by attention and trips to resorts. When last seen three months ago, the patient was in good health and had a good knee. I am informed that she has no pain except in cloudy weather. Undoubtedly impaired alimentary and tissue digestion associated with faulty elimination, was the basis of the condition. The removal from the joint of the enlargement and offending tissues permitted it to share in the restoration of health.

CASE 2. Seen first in February, 1902. She had been subject to "chronic rheumatism" in the left knee for six years. It came on insiduously with gradual swelling, pain and tenderness. She went to Hot Springs a number of times, and had taken various antirheumatic remedies since the knee first began to trouble her.

The only local treatment the joint had had was a plaster paris cast which was worn one year. There was no improvement from this treatment. Examination showed the leg to be flexed at the knee about twenty-five degrees. Flexion was possible to about sixty degrees. The joint was swollen. The swelling was uniform over the whole joint. In the upper outer quadrant a thickening could be felt. If one placed the index finger and thumb on either side of the patella and the leg was flexed and extended alternately one felt a mass slide beneath the fingers. The joint was tender to touch all over, chiefly internal to the patella tendon. The knee pained all the time. At night sleep was interfered with. Sometimes she was in bed several days on account of the pain. She was very lame. She was put to bed about two weeks. Hot fomentations were applied to the knee. The joint was then opened on both sides. The synovial and fibrous capsule was thickened. This tissue was tough and fibrous. The external condyle of the femur showed an eroded area about the size of a quarter. The capsule and thickened tissue, part fat and part fibrous tissue, were removed from the side of and beneath the patella tendon. The lipomatous tissue in the upper outer quadrant was removed, joint was flushed out and closed. It has been four years since this operation. This lady to-day is able to walk all day shopping and goes where she pleases. She limps, not from pain, but on account of the fact that the leg is shorter than its fellow. The flexion is reduced to about five degrees. She can voluntarily flex the leg on the thigh about sixty-five degrees.

CASE 3. This patient came under observation two years ago complaining of "rheumatic gout," and stated that she had been told nothing could be done for her. She weighed two hundred and thirty pounds. Her knees were large and so fat that nothing could be palpated. They were exceedingly tender, the least pressure on the knee-pan causing pain. The knees ached so badly that she could hardly sleep at night. Her general condition was bad.

She had headaches daily and dyspepsia. The feet and ankles were very tender. She had at times pains in the other joints. She could not go up and down the stairs. She could not walk across the house except with a great deal of pain. She could not attend to her household duties. The knee joints were opened and from the inner and outer upper quadrants of each knee small lipomata were removed. These lipomata consisted of thickened trabeculæ of fibrous tissue including lobules of fat. The lower quadrants of the knees presented nothing abnormal.

She made a good recovery from the operation. Before the operation in spite of the rigid dieting and the administration of diuretics, antirheumatics and purgatives, the pain persisted. Since the operation dieting has so improved this patient that she now walks a mile a day with absolutely no discomfort, and has absolutely no difficulty whatever in attending to her household duties. She has to wear foot-plates. For years she has been given drugs to remove the pain from the knees, which were painful mainly from the presence of the lipomata. Naturally no relief came until these small tumors had been removed. It is interesting to note that after the lipomata had been removed, at times pain in the knees would recur, and that then, since the joints had been freed from the presence of these damaging bodies, dieting and antirheumatic remedies brought relief.

Gradually by carefully regulated life the rheumatic tendency was conquered by her family physician, so that only now and then do the joints give pain, and then only slightly. These lipomata in the knees are not uncommon. This patient has the normal amount of motion in the knee-joint.

Case 4. Miss L. This lady has been subject to "chronic rheumatism" for seventeen years. She had made yearly trips to Hot Springs. The baths there had undoubtedly helped her to keep down her rheumatic condition but had had little influence upon the knee joints. About a year ago while attempting to cross her knees, the right one became locked, and she could not unlock it. The knee was not any more painful than before the locking happened, but it pained a great deal. She had been able to walk a short distance without using a cane, but she usually used a cane and had been a constant though cheerful sufferer. The joint was opened two days after she was seen April 10, 1905. This joint was filled with very succulent fringes. The cavity was filled up with these fringes and small polypi hanging from the synovial surface of the capsule. The fringes and polypi were excised. In the upper quadrant, tumors about two inches long and one-half inch wide were removed. The fixation of the joint was due to the fact that two bone spurs were interlocked in the act of flexing the leg. These spurs, one of the femur and one of the tibia, were of course, removed. Figure one shows a skiagraph of this knee.

FIG. 1.

She made a good recovery from the operation. June 15, 1905, when last seen, she walked without crutch or cane. She wore an apparatus for some time because there was a tendency for the hamstring muscle to hold the leg in permanent flexion of about ten degrees. No additional motion was gained by the operation but her locomotion had been vastly improved and the pain decreased.

CASE 5. Mc. W. She had an attack of "rheumatism" two years ago, lasting six weeks in which both knees, one elbow and one hip were affected. All the joints recovered but the knee. When first seen, May 15, 1905, the knee was very much swollen as is shown in Figure 2.

FIG. 2.

The capsular space was very much enlarged by the distention several inches above the patella. The joint was opened. A quantity of straw-colored fluid evacuated. A round fatty tumor, "a" in Figure 3, was floating unattached in the joint. The upper quadrants of the sack presented tuberous growths, fatty and fibrous in character, attached by broad bases to the capsule. The capsule proper was so infiltrated with this tissue that the uppermost part of it was excised with the growths. The cut edges were sutured beneath the quadriceps tendon so that the capsule was complete though smaller after excision of the upper portion. Figure 3 shows the fatty tumors and polypi removed from the joint. The ends of the femur and tibia were normal. Figures 4 and 5 show the appearance standing and the motion in flexion four months after the operation. The patient, in so much as function is concerned, has a normal joint. There is no pain and no limitation of motion.

FIG. 3.

CASE 6. "Chronic rheumatism" for several years. Both knees were painful. One and one-half years ago she began to have severe sciatic pains. She noticed that "little things" could be felt beneath the skin over the joints. The "little things" appeared first in one part of the joint and then the other. The joint was opened by another surgeon, and a number of small cartilaginous bodies from one-fourth to three-fourths inches long were washed out of the joint. This relieved the sciatic pain largely. She made recovery from that operation. Three months after she begun to walk, again she had sharp attacks of pain in the left knee which would necessitate rest for the leg for two or three days. These attacks continued. They would come on at any time in bed, or walking but there was always associated with the flexion rotation in the joint. The joint was opened. A small cartilaginous body about three-fourths by one-fourth inch in size was found attached to the anterior face of the capsule between the patella, femur and tibia. This body hung attached by a pedicle the size of a match and one-half inch long. The joint surfaces, cartilaginous and capsular, were injected, and numerous spurs protuberated from the cartilaginous margins of the femur. Three of these were of sufficient size to possibly produce trouble at some future time and were removed. The internal semilunar was excised.

This patient was operated upon four months ago. The operation will have no effect upon the diathesis but the sharp attacks have not recurred.

FIG. 4 AND FIG. 5.

These few cases are indicative of the many conditions, the result of inflammations, toxic, rheumatic or whatever one may at present call them. A limited space permits me to report only so many as may suffice to bring out the point to which I desire to forcibly call your attention. In continued "rheumatic" inflammation of the knee joint, if there be swelling and lameness, there are two things to command attention, viz.: the diathesis, and the above-mentioned pathological formations. Such a joint requires operation and apparatus often subsequently in addition to diet, hygiene, hydrotherapy and the administration of medicines in order to produce the best results possible for the individual patient.

Only by this combined attention can the best possible results be obtained.

DISCUSSION OF DR. HOKE'S PAPER.

*Dr. Barnett:* I was unfortunate in not being able to hear all of Dr. Hoke's paper, but I have had the pleasure of following him in his operative work. I of course do not know what condition existed prior to operations or their future history, but saw most of them only at time of operation. I have seen knees made flexible which could not be flexed. I have seen joints opened with perfect safety, that is, with as much safety as opening the abdomen. I am certain that myself and others have treated some of these cases wrong, whereas, in the new light we have before us, we would treat otherwise.

*Dr. Block:* It would be interesting to know the cause of the growth of these fatty tabs in the knee-joint. We know that passive congestion causes an increased growth of bone, and we believe also of connective tissue. It would be interesting to know if this is also true of fat. Osteoplytes and exostoses can be produced experimentally in rabbits by the frequent and prolonged application of a ligature for twenty minutes at a time.

It is a remarkable fact that the only cases of fatty tabs that I have heard of, interfering with the motion of joints, have all been in the knee joint. I would like to ask if they ever occur in other joints?

*Dr. Cartledge:* I would like to ask if the genito-crural nerve has anything to do with these cases, as most of the patients seem to be females.

*Dr. Ballenger:* I would like to know if any of these cases operated on by Dr. Hoke were found to be gonorrheal.

*Dr. Hoke* (in closing): Dr. Block asks why the condition is more common with women than men, and if the soft tissue growths occur in the finger joints? Undoubtedly the various catarrhs are prominent factors in these cases. Many of the knee-joint cases are infectious in origin. Women are frequent sufferers from vaginitis, inflammation of the uterus and its appendages, etc. These inflamed areas are often the source of the infection. Then, too, their sedentary lives makes them more liable to the diathesis as well as to infection.

The difference in the condition of the soft tissues in and around the swollen joints from that found in the knee-joints is due to the difference in anatomical structure. Fibrous tissue thickening is always present, bone spurs are common, diverticula from the capsule which cover the spurs are cemmon, but the fringes, polypi and fatty tumors are not found, owing to the lack of an anatomical basis for this formation and a large cavity in which they may grow. None of these cases were gonorrheal in origin.

## Figures and Tables

**Fig. 1. f1:**
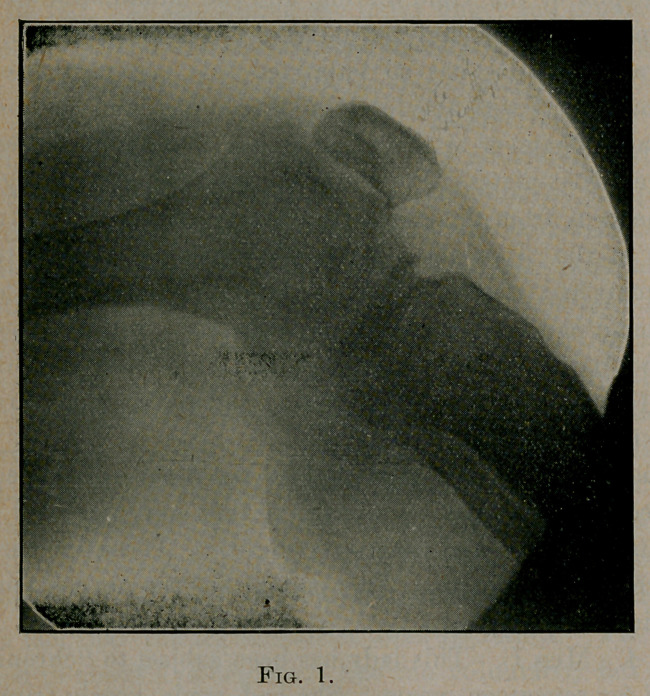


**Fig. 2. f2:**
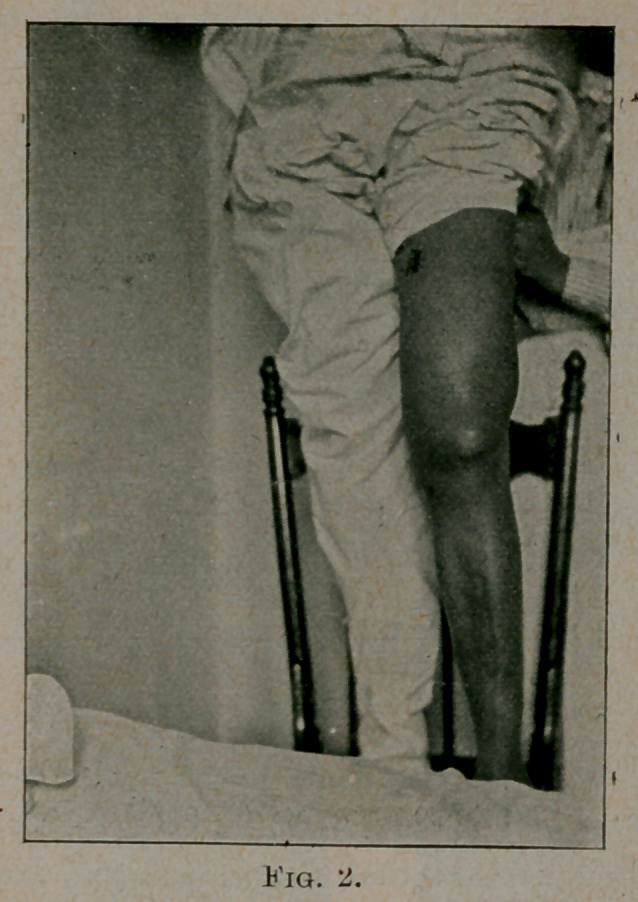


**Fig. 3. f3:**
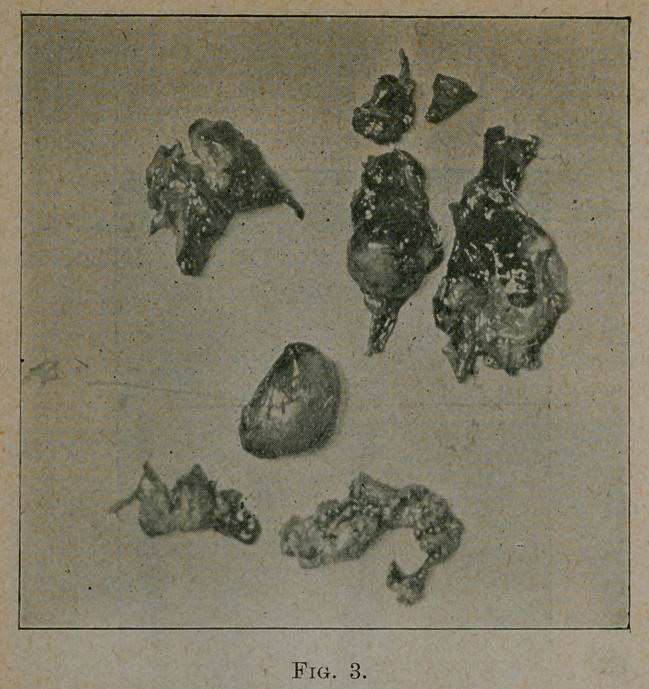


**Fig. 4 and Fig. 5. f4:**